# Longitudinal functional lung imaging in children with Post-COVID-19 syndrome

**DOI:** 10.1186/s40348-025-00216-x

**Published:** 2026-01-31

**Authors:** Calvin Kraus, Lina Tan, Maximilian Hinsen, Sandy Schmidt, Emmanuel Nedoschill, Felix Wachter, Henriette Mandelbaum, Alexandra L. Wagner, Isabelle Schöffl, Annika Weigelt, Manfred Rauh, Joachim Woelfle, Michael Uder, Regina Trollmann, Jens Vogel-Claussen, Adrian P. Regensburger, Rafael Heiss, Ferdinand Knieling, Roman Raming

**Affiliations:** 1 Department of Pediatrics and Adolescent Medicine, University Hospital Erlangen, Pediatric Experimental and Translational Imaging Laboratory Translational Pediatrics, Loschgestraße 15, Erlangen, 91054 Germany; 2 University Hospital Erlangen, Institute of Radiology, Erlangen, Germany; 3 Department of Pediatric Neurology, Center for Chronically Sick Children, Charité, Berlin, Germany; 4 Department of Pediatric Cardiology, University Hospital Erlangen, Erlangen, Germany; 5 Institute for Diagnostic and Interventional Radiology, Hannover Medical School, Hannover, Germany

**Keywords:** Post-COVID, SARS-CoV-2, Functional lung imaging, PREFUL MRI, Pediatric

## Abstract

**Background:**

Respiratory distress and COVID-19-related symptoms persist as Post-COVID-19 syndrome (PCS) in a proportion of children and adolescents.

We aimed to determine whether ventilation or perfusion defects constitute a possible cause for PCS.

**Results:**

Mean ventilation was lower at baseline in vaccinated than in PCS (p=0.04) and V/Qmatchnon-defected increased from baseline to follow-up in vaccinated (p=0.03). In post-hoc comparison with historic data, V/Qmatchnon-defected improved in recovered (historic: 63.5±18.7%; baseline: 88.8±9.0%, p<0.0001; follow-up: 88.1±10.6%; p=0.0002) and PCS (historic: 57.3±19.5%; baseline: 86.1±7.6%, p<0.0001; follow-up: 86.6±7.7%, p=0.0110). Symptom load in PCS decreased from infection and baseline time points to 6-month follow-up. Laboratory assessments showed no differences.

**Conclusion:**

Despite persisting clinical symptoms, PREFUL MRI demonstrates no significant difference between cohorts, not supporting the hypothesis of persisting defects of the lung as causative.

**Supplementary Information:**

The online version contains supplementary material available at 10.1186/s40348-025-00216-x.

## What is known

- Respiratory distress and COVID-19-related symptoms persist as Post-COVID-19 syndrome (PCS) in a proportion of children and adolescents.
- COVID-19 is primarily a respiratory disease.

## What is new

- Longitudinal PREFUL MRI (phase-resolved functional magnetic resonance lung imaging) demonstrates no significant difference between cohorts, not supporting the hypothesis of persisting defects of the lung as causative.

## Introduction

As of February 2025, the severe acute respiratory syndrome coronavirus type 2 (SARS-CoV-2) pandemic was attributed to more than 777 million cases and seven million deaths [1]. The introduction of vaccines in late 2020 led to a reduction in overall mortality and patients requiring intensive care [2, 3], shifting the focus toward the long-term sequelae of the infection [4, 5]. Post-COVID-19 condition (PCC) as defined by the WHO [6], or equivalently Post-COVID-19 syndrome (PCS) according to the German S1 guideline (AWMF) [7], refers to any symptoms that develop within three months of a confirmed or probable acute infection and persist for at least two months without being explained by an alternative diagnosis. The most common symptoms include fatigue [4, 8, 9], dyspnea [10–12], cognitive dysfunction [13, 14], and exercise intolerance [4, 11, 12, 15]. While most children and adolescents recover quickly from acute infection with SARS-CoV-2 and are generally less affected [16, 17], growing evidence indicates that a subset of patients experiences prolonged symptoms also at a younger age [4, 8–12, 15, 18]. Estimates for the prevalence of PCS range from 6 to 15% for the general population [19, 20]; however, in children the prevalence appears to be lower, at about 2 to 3.5% [21]. Until now, precise numbers are missing [22–24]. Another challenge is the availability of suitable methods to objectively measure and monitor symptoms. Advanced lung imaging modalities have shown promise in visualizing and quantifying pulmonary dysfunction in adults and children [25–29]. In this regard, phase-resolved functional lung (PREFUL) low-field magnetic resonance imaging (LF-MRI) may provide a radiation-free method applicable to children and adolescents during free breathing [29].

In this study we longitudinally investigated children vaccinated prior to infection with SARS-CoV-2, children who had recovered from acute infection and compared them to patients with PCS. Additionally, we performed an exploratory comparison of the results with historic data to derive long-term information regarding the differences in healthy and PCS participants [29] and report on clinical development until over 900 days after infection .

## Methods

### Study design and setting

Between July 2022 and October 2023, we conducted a prospective, open-label, observational, single-center study to investigate lung parenchymal changes in children and adolescents after SARS-CoV-2 infection over the course of six months (clinicaltrials.gov ID NCT05445531). Randomization wasn’t intended. Assignment to cohorts was based on presence or absence of disease. As far as possible, specialist departments performing tests were blinded to clinical features by study directors. The study complied with the Declaration of Helsinki and was performed according to ethics committee approval (no. 22-77-Bm). All parents or guardians, and, if appropriate, participants gave written informed consent to participate in the study and to publication of the images in Figs. 2a and 3a.

The call for study participation was nationwide and publicly announced using our clinic’s and university’s online presence. The responding participants were screened for eligibility to one of three study cohorts based on their age (5 to < 18 years), time since SARS-CoV-2 infection, presence of major disabilities and continued interest in participating. Screening was performed through direct outreach via email or telephone. Cohorts were defined as follows: vaccinated participants were required to have received at least two doses of a European Union-approved vaccine at least two weeks prior to their first COVID-19 infection to ensure a likely sufficient immune response. Recovered participants were those who were either unvaccinated or incompletely vaccinated at the time of acute COVID-19 but did not show prolonged symptoms. Participants with PCS at baseline were included regardless of their vaccination status. PCS was defined according to the German S1 guideline at the time of inclusion [7], matching the criteria of the WHO definition of Post-COVID-19 condition (PCC) [6]. Inclusion criteria were: age 5 to < 18 years, proof of at least two vaccine doses for the vaccinated cohort, proof of SARS-CoV-2 infection for the recovered and PCS cohort, and fulfillment of PCS criteria according to the German S1-guideline for the PCS cohort. Exclusion criteria consisted of ages outside the predefined range, acute SARS-CoV-2 infection and a need for isolation or quarantine measures, acute infections in general, pregnancy, any critical medical condition, the refusal to undergo MRI and any contraindications to MRI, electrical implants such as pacemakers or perfusion pumps.

The sample size was based on expected changes in the PCS group. At a statistical power of 0.80, an alpha of 0.05 a mean of 60% V/Qmatch_non−defected_ (ventilation perfusion match of non-defected areas) at timepoint baseline (0 months) (µ_1_) and at timepoint follow-up (6 months) of 70% was expected. With a σ of 20 and a ∆ of 10 yields itself a number of cases of 33 per cohort. At a dropout rate of 10%, 37 participants were needed.

After inclusion, participants were assessed at two independent timepoints: baseline (0 months) and follow-up (approximately six months). Baseline appointment was conducted over the course of several weeks, given the availability of both participant and researcher. The Follow-up appointment was then planned to match the six-month-interval relative to the date of first appointment, average interval was 215 days ± 47 days. At their first appointment participants and/or their parents were polled about their medical history, followed by a clinical assessment, drawing of blood samples and PREFUL MRI of the lung. Clinical characteristics and imaging results of the three cohorts were compared between these two timepoints.

For post-hoc comparison, a subset of datasets was included to evaluate long-term effects and are hereby referred to as “historic”. Out of the participants of a previous, cross-section study (clinicaltrials.gov ID NCT04990531), which also implemented Low-Field-MRI, laboratory and clinical assessment, a number of participants (recovered: *n* = 5; PCS: *n* = 3) volunteered to participate again in our longitudinal assessment. Therefore, for this subset, three timepoints exist (historic, baseline and follow-up) which were evaluated longitudinally using the same parameters as described before to further identify changes over time in this subset.

### Clinical data and blood samples

All participants were assessed for medical history, symptoms during and after COVID-19 infection and immunity status for SARS-CoV-2. Height, body weight, blood pressure and heart rate were measured for each patient using in-house standard equipment. At each time point a blood sample was collected to assess a standard blood count, interleukin-6 (IL-6) and C-reactive protein (CRP) as common markers of inflammation, and antibodies against SARS-CoV-2 for laboratory confirmation of SARSCov2-infection. Further details are available in the Supplementary Appendix.

Clinical symptoms, both retrospectively reported at initial infection and currently at baseline and follow-up, were assessed at each study visit using a predefined questionnaire. Depending on the age of the participants, symptoms were reported by the children themselves and/or the parents, serving as third-party anamnesis. Primary symptoms were headache, cold, sore throat, cough, dyspnoea, pneumonia, fever, loss of scent, loss of taste, fatigue and melalgia. Secondary symptoms were clustered in gastrointestinal, neurological, psychological and other symptoms, as well as susceptibility to infection and susceptibility to inflammation. Diarrhea, nausea and vomiting, and abdominal pain were clustered as gastrointestinal symptoms; sight and sleeping disorder, tinnitus, paresthesia, phantosmia, absence seizure and aphasia as neurological symptoms; depression, forgetfulness and weak concentration as psychological symptoms and undefined chest pain, sensitivity to low temperatures, cramping fingers and recurring hematoma as other symptoms. For statistical analysis, symptom load was compared as the percentage of the respective cohorts affected by the specific symptom or cluster.

### Magnetic resonance imaging

The participants underwent low-field magnetic resonance imaging (LF-MRI, 0.55 Tesla Magnetom Free.Max, Siemens Healthineers, Erlangen, Germany) of the thorax to assess ventilation and perfusion of the lung and morphological changes. As described previously [29], a standard body coil was used for free-breathing lung imaging. No sedation was used in the acquisition of the images; all participants followed a short breathing instruction and continued breathing freely. The final image used for interpretation was a single two-dimensional coronal image aligned at the center of the lung hila with a thickness of 15 mm, an in-plane resolution of 2.0 × 2.0 mm giving a matrix of 128 × 128 (interpolated to 256 × 256), with a bandwidth of 1395 Hz per pixel, a flip angle of 30°, using a repetition time/echo time of 231.3/1.3 ms respectively, a parallel imaging acceleration factor of 2, no partial Fourier, measuring a total of 388 time points, giving a temporal resolution of 232 ms, acquiring all this during an examination time of 1 min and 30 s [29].

For analysis of PREFUL MRI, dedicated software (Siemens Healthineers, Germany) was used to calculate the following parameters voxel-wise after automatic registration and manual confirmation to a midexpiration position. The following parameters were calculated: mean ventilation (expressed as percentages), calculated as the difference between signal values at a neutral middle lung position in relation to end-inspiration and end-expiration values; and mean perfusion (as percentages) with respect to a full-blood signal such as the aorta, vena cava or ventricles; and flow-volume loop correlation, defined as ratio between flow-volume loop, deducted from reconstructed ventilation cycle, and the largest connected region within the 80th and 90th ventilation percentiles. Subsequently defect areas in percent were calculated for ventilation (VDP) and perfusion (QDP) based on a large previous sample. Areas with or without defects were calculated by forming the ratio of ventilation to perfusion, resulting in V/Qmatch for both defected and non-defected areas. V/Qmatch_non−defected_ was used to represent the percentage of healthy lung tissue in the measured frontal plane. PREFUL MRI and its parameters were validated using dynamic contrast-enhanced MRI [30–32], ^129^Xenon ventilation MRI [33], and V/Q SPECT, as well as being shown to be repeatable regarding ventilation and perfusion [34], to correlate to HP ^129^Xe MRI in Pediatric Cystic Fibrosis [35], and to correlate to spirometry in asthma [36]. The evaluating radiologist was blinded to clinical features for all analyses.

### Statistical analysis

Continuous variables are given as means with standard deviation and categorical variables as numbers with percentages. The standard deviation was calculated using the standard formula with Bessel’s correction (n-1). A nonparametric Kruskal-Wallis test with corrected Dunn test for post hoc comparisons between groups was used to assess differences in vaccinated or healthy controls, participants who recovered from COVID-19, and participants with PCS. In cases with only two compared cohorts a Mann-Whitney test was used. Additionally, a Wilcoxon matched-pairs signed-rank test was used for the subset of participants with follow-up data. P values are reported. Prism 10, version 10.2.3 (GraphPad Software) was used for all statistical analyses. *p* ≤ 0.05 was considered to indicate statistically significant difference in all analyses.

### Illustration

For illustration BioRender and Adobe Illustrator 2025 Mac 29.6.1 was used. All rights on BioRender made figures are cited below regarding the respective figures.

**Fig. 1:**
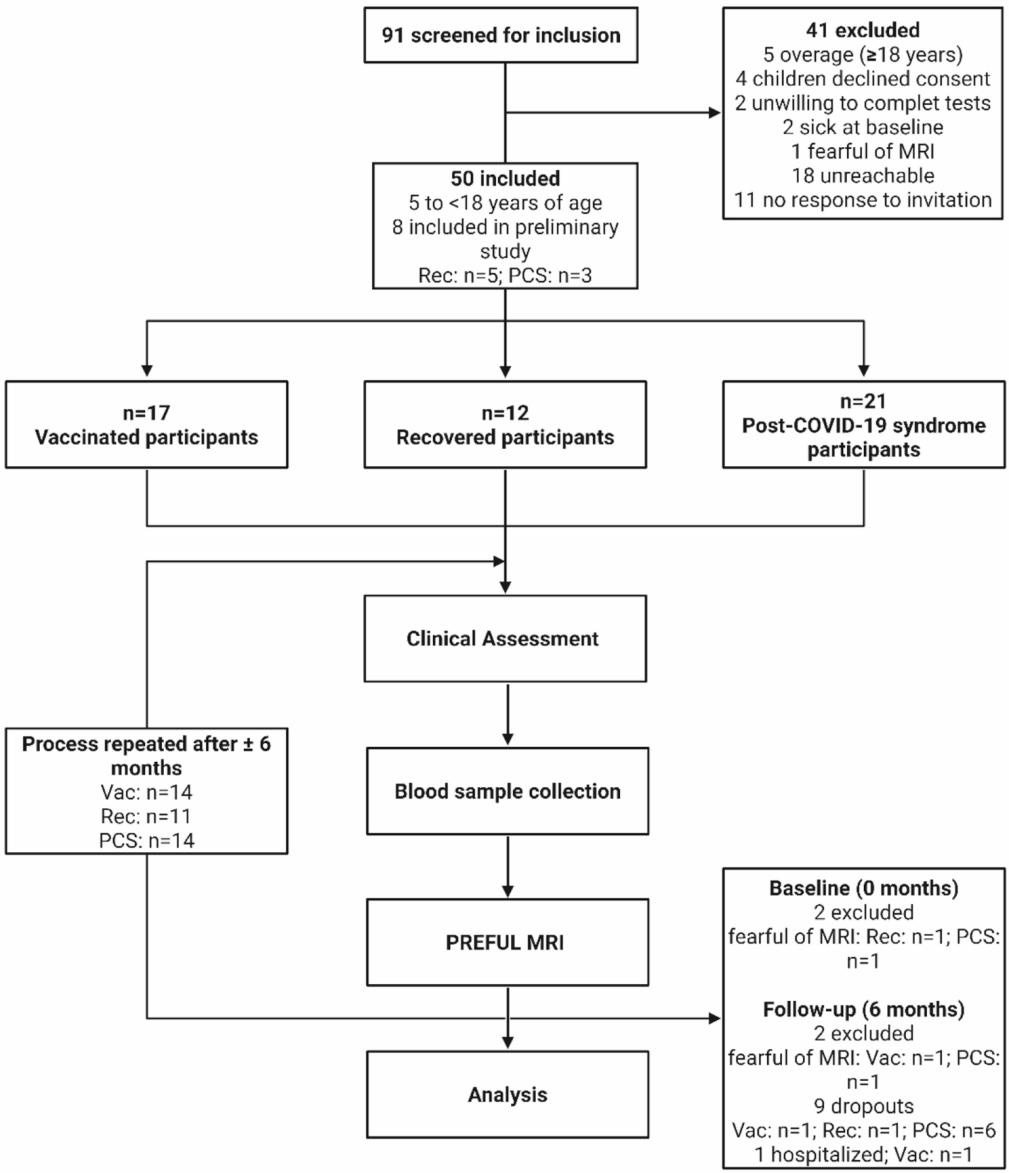
Study flow chart. Participants were screened for inclusion, assigned to three cohorts based on definition of Post-COVID-19 syndrome by the WHO [6]. A subset could be recruited for a follow-up, repeating the process. Vac = Vaccinated; Rec = Recovered; PCS = Post-COVID-19 syndrome; PREFUL MRI = Phase-resolved functional lung low-field magnetic resonance imaging

Figure 1: Created in BioRender. Kraus, C. (2025) https://BioRender.com/eev79x4.

**Fig. 2:**
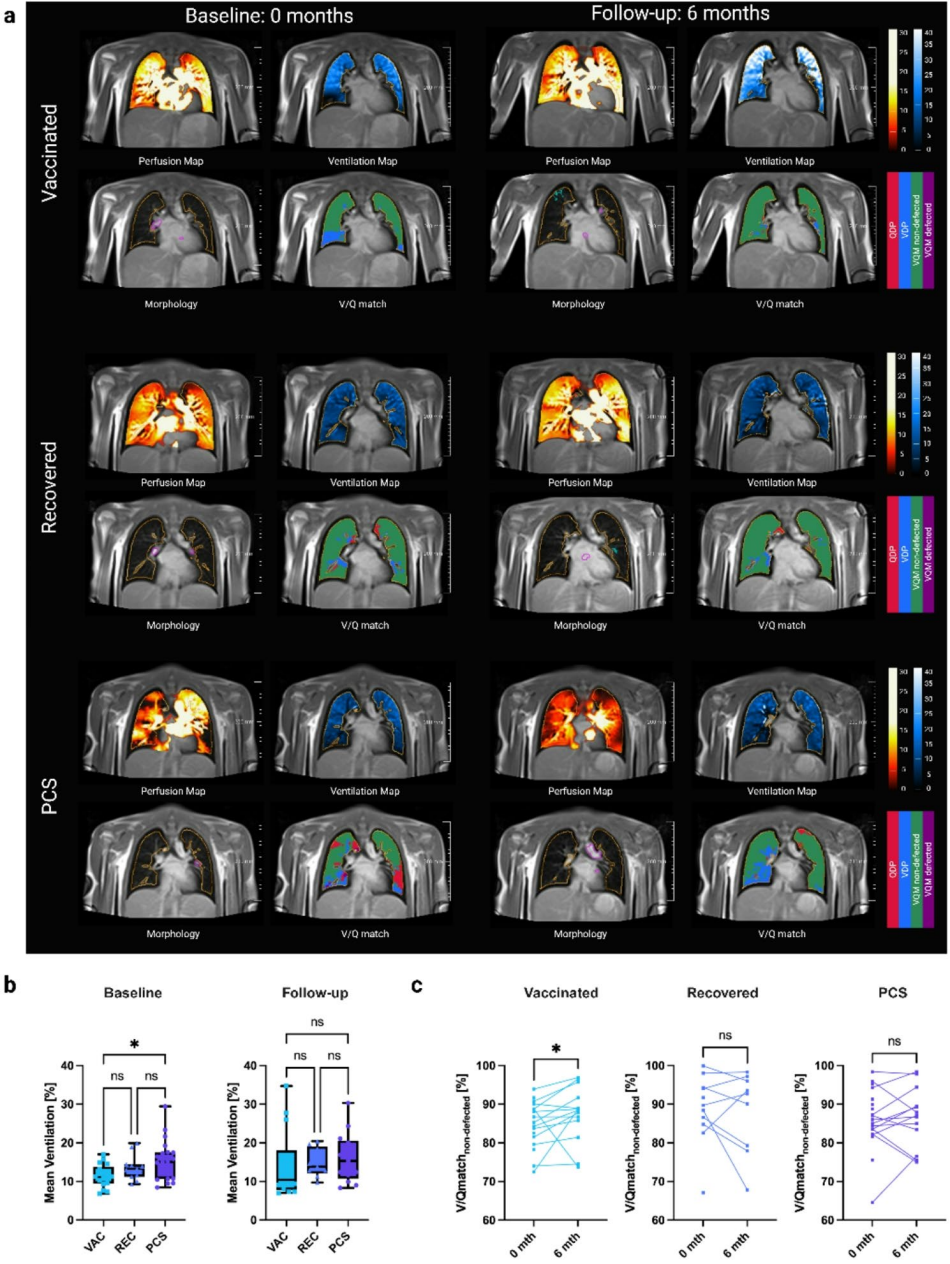
Representative PREFUL MRI scans at baseline and 6-month follow-up. **a **Representative PREFUL MRI scans of a vaccinated, recovered and Post-COVID-19 syndrome participants at baseline and 6-month follow-up. For each timepoint a perfusion, a ventilation map, morphology and ventilation/perfusion match are displayed. Provided scales are 200mm. **b **Comparisons of vaccinated, recovered patients, and PCS patients. Vaccinated showed lower mean ventilation than PCS, at follow-up ventilation of vaccinated had increased and no difference remained between cohorts. P values were assessed with Kruskal-Wallis test. **c** From baseline to follow-up vaccinated showed increased V/Qmatchnon-defected. P values were assessed with Wilcoxon matched-pairs signed rank test. Vac = Vaccinated, Rec = Recovered, PCS = Post-COVID-19 syndrome, PREFUL MRI = Phase-resolved functional lung low-field magnetic resonance imaging, V/Qmatchnon-defected = ventilation perfusion match of non-defected areas. Asterisks represent significant differences. *P ≤ 0.05

Figure 2: Created in BioRender. Kraus, C. (2025) https://BioRender.com/i6t2mp7.

**Fig. 3:**
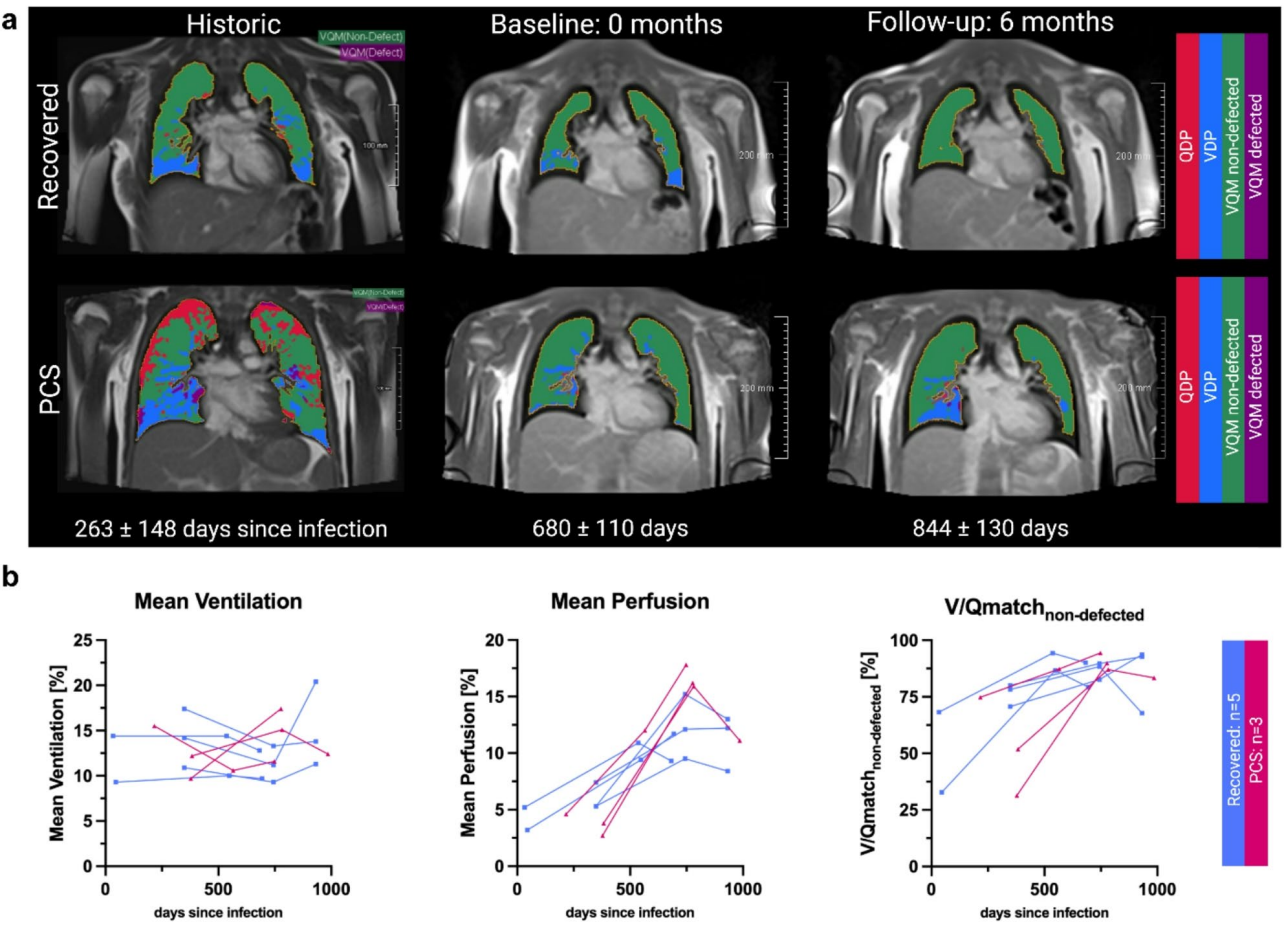
Representative long-term PREFUL MRI assessments. **a** Representative images of V/Qmatch derived from PREFUL MRI of a recovered and a PCS participant followed over 844 days after initial infection. Historic data is derived from a preliminary study [29]. Provided scales are 100mm for historic and 200mm for baseline and follow-up. **b** Connected dot plots show trends for ventilation, perfusion and V/Qmatchnon-defected in recovered and PCS cohorts. Rec = Recovered, PCS = Post-COVID-19 syndrome, PREFUL MRI = Phase-resolved functional lung low-field magnetic resonance imaging, QDP = Perfusion defect percentage, VDP = Ventilation defect percentage, V/Qmatch = Ventilation-perfusion match

Figure 3: Created in BioRender. Kraus, C. (2025) https://BioRender.com/kax5i6d.

## Results

### Patient characteristics and clinical standard assessments

A total of 91 children and adolescents were screened for inclusion, of whom a total of *n* = 50 were included. They were divided into cohorts of *n* = 17 vaccinated, *n* = 12 recovered, and *n* = 21 PCS patients. Six months after initial visit, *n* = 14 vaccinated (Vac), *n* = 11 recovered (Rec), and *n* = 14 PCS were re-assessed (Fig. 1). None of the participants with COVID-19 required hospital admission during the primary infection period. The mean interval between positive SARS-CoV-2 RT-PCR test and study participation was 328 ± 225 days (Vac: 178 ± 66 days, Rec: 429 ± 231 days, PCS: 342 ± 240 days). The demographic data are given in Table 1. At baseline heart rate was lower in vaccinated than in recovered and PCS (Vac to PCS *p* = 0.0180; Vac vs. Rec *p* = 0.0081; Rec vs. PCS p = ns). Time from 2nd vaccination to infection was longer in vaccinated than in recovered (*p* = 0.0464). Additionally, time from infection to examination was shorter in vaccinated participants with prior infection compared to recovered participants at both baseline (*p* = 0.0174) and follow-up (*p* = 0.0100). Preexisting conditions were found in 11.8% of vaccinated and 52.4% of PCS participants, while recovered had no preexisting conditions. Two vaccinated participants and one PCS participant reported depressive disorders, one vaccinated panic disorders, four PCS each reported asthma or allergies, two PCS allergic rhinitis, and one PCS strabismus. The most common symptoms after infection are given in Supplementary Fig. 1a. The PCS cohort reported persisting symptoms 12 weeks after initial infection. Most participants reported fatigue (52.4%), followed by dyspnea (42.9%) and headache (28.6%). At follow-up 210 ± 47 days later, 5 out of 14 participants in the PCS cohort (35.7%) still showed persisting symptoms. This time dyspnea (21.4%) was the leading symptom. At baseline and follow-up 190 days later, one participant (10.0% and 12.5%) in the vaccinated cohort reported persisting headache after infection and vaccination. In the recovered cohort, one participant (8.3%) reported persisting dyspnea and another (8.3%) persisting neurological symptoms after infection, although stating not to suffer from prolonged symptoms (Supplementary Fig. 1a). For a complete list of symptoms see Supplementary Table 1.

**Table 1:** Patient demographics

	Vaccinated		Recovered		PCS	
Examination	Baseline (*n* = 17)	Follow-up (*n* = 14)	Baseline (*n* = 12)	Follow-up (*n* = 11)	Baseline (*n* = 21)	Follow-up (*n* = 14)
Age [y]	12.8 ± 2.8	13.2 ± 2.7 *	10.3 ± 3.9	11.4 ± 3.5 *	11.4 ± 3.5	13.7 ± 2.5 **
Weight [kg]	50.1 ± 16.4	53.6 ± 16.8 ***	44.8 ± 24.2	50.2 ± 24.3 ***	51.4 ± 14.6	54.9 ± 14.6 *
Height [cm]	160.3 ± 17.4	163.6 ± 17.4 ***	149.7 ± 20.3	154.7 ± 18.4 *	157.0 ± 13.0	161.5 ± 12.8 **
BMI [kg/m^2^]	19.0 ± 3.2	19.5 ± 3.2 **	18.7 ± 5.6	19.9 ± 5.6	20.0 ± 4.3	20.9 ± 4.6
Sex						
F^a^	7 (41.2)	4 (28.6)	8 (66.7)	8 (72.7)	11 (52.4)	8 (57.1)
M^a^	10 (58.6)	10 (71.4)	4 (33.3)	3 (27.3)	10 (47.6)	6 (42.9)
Race or ethnic group						
Caucasian^a^	17 (100)	14 (100)	12 (100)	11 (100)	20 (95.2)	13 (92.9)
Arabic^a^	0 (0)	0 (0)	0 (0)	0 (0)	1 (4.8)	1 (7.1)
Vital parameters						
Systolic blood pressure [mmHg]	113.1 ± 11.0	117.7 ± 12.3	123.5 ± 12.2	118.0 ± 7.7 *	116.6 ± 11.5	120.0 ± 13.8
Diastolic blood pressure [mmHg]	61.6 ± 7.9	62.4 ± 8.7	65.5 ± 7.2	66.7 ± 8.8	65.7 ± 8.3	66.7 ± 8.2
Heart rate [/min]	76.0 ± 10.5	81.1 ± 9.5 *	92.8 ± 13.7	92.9 ± 14.1 *	89.7 ± 16.9	96.1 ± 23.7
Medical history						
Preexisting conditions	2 (11.8)		0 (0)		11 (52.4)	
Depressive disorders	2 (11.8)		0 (0)		1 (4.8)	
Panic disorders	1 (5.9)		0 (0)		0 (0)	
Bronchial asthma	0 (0)		0 (0)		4 (19.0)	
Allergic rhinitis	0 (0)		0 (0)		2 (9.5)	
Allergies	0 (0)		0 (0)		4 (19.0)	
Strabismus	0 (0)		0 (0)		1 (4.8)	
Infection / Vaccination / Examination						
Infection^a^	10 (58.8)	9 (64.3)	12 (100)	11 (100)	21 (100)	14 (100)
Infection to examination (d)	178 ± 66	376 ± 103	429 ± 231	662 ± 200	342 ± 240	586 ± 242
2nd infection^a^	1 (5.9)	1 (7.1)	1 (8.3)	1 (9.1)	3 (14.3)	3 (21.4)
2nd infection to examination (d)	143	356	129	342	288 ± 69	459 ± 34
2 vaccinations prior to infection^a^	10 (58.6)	9 (64.3)	2 (16.7)	2 (18.2)	13 (61.9)	8 (57.1)
2nd vaccination to 1st infection (d)	166 ± 116		7 ± 7		109 ± 77	
2nd vaccination to examination (d)	333 ± 83	571 ± 104	292 ± 121	488 ± 119	310 ± 93	556 ± 107
baseline to follow-up (d)		229 ± 43		204 ± 52		210 ± 47

Data are means ± SD, unless otherwise specified

*F* Female, *M* Male, *PCS* Post-COVID-19 syndrome

^a^ Data are numbers of participants, with percentages in parentheses

* Adjusted P-values were calculated using the Wilcoxon matched-pairs signed rank test to compare baseline and follow-up. Asterisks represent significant differences between baseline and follow-up. *P ≤ 0.05 **P≤0.01 ***P≤0.001

When comparing the symptom load, defined as the proportion of the cohorts affected by the predefined symptoms or symptom clusters, no significant difference between cohorts was found at initial infection. At baseline the PCS cohort had a higher symptom load than recovered and vaccinated (Vac: 0.6 ± 2.4%, Rec: 0.9 ± 2.7%, PCS: 17.2 ± 15.0%; Vac vs. Rec: p = ns; Vac vs. PCS: *p* < 0.0001; Rec vs. PCS: *p* < 0.0001). At follow-up, the symptom load remained higher in PCS compared to recovered and vaccinated (Vac: 0.7 ± 2.9%, Rec: 0.0 ± 0.0%, PCS: 4.4 ± 6.5%; Vac vs. Rec: p = ns; Vac vs. PCS: *p* = 0.0173; Rec vs. PCS: *p* = 0.0037) (Supplementary Fig. 1b, Supplementary Table 1). For the PCS cohort, symptom load decreased from baseline to follow-up (*p* = 0.0001) (Supplementary Fig. 1c).

### Laboratory assessments

In laboratory assessments, no differences were found between cohorts or timepoints regarding mean hemoglobin, thrombocyte and leukocyte count, CRP, and IL-6 (Supplementary Table 2). Out of the 36 children and adolescents with successfully drawn blood samples, everyone showed positive SARS-CoV-2-spike protein antibodies, and only two vaccinated participants (15.4%) were negative for nucleocapsid antibodies (COI < 1.0) indicating no previous exposure to SARS-CoV-2.

### Functional lung imaging

The PCS group showed higher mean ventilation than the vaccinated group at baseline (Vac to PCS *p* = 0.04; Vac vs. Rec p = ns; Rec vs. PCS p = ns), but not at follow-up (Fig. 2b). When comparing baseline to follow-up within each cohort, no differences were found (Supplementary Fig. 2b). No significant difference in V/Qmatch_non−defected_ was found among the groups (Vac: 84.7 ± 6.1%; Rec: 88.8 ± 9.0%; PCS: 86.1 ± 7.6%) (Supplementary Fig. 7a). Values only increased in the vaccinated cohort from baseline to follow-up (Vac: *p* = 0.03; Rec: p = ns; PCS: p = ns) **(**Fig. 2c**)**. Regarding mean perfusion, V/Qmatch_defected_, VDP and QDP, no differences were found either between the vaccinated and the COVID-affected cohorts or between baseline and follow-up (Supplementary Fig. 3–6). For a complete imaging list displaying combined V/Q defects, see Table 2.

**Table 2:** PREFUL MRI quantifications at baseline and 6-month follow-up

	Vaccinated		Recovered		PCS	
Examination	Baseline (*n* = 17)	Follow-up (*n* = 14)	Baseline (*n* = 11)	Follow-up (*n* = 9)	Baseline (*n* = 20)	Follow-up (*n* = 13)
Mean ventilation (%)	11.5 ± 2.9	14.1 ± 8.8 *	13.6 ± 3.3	14.8 ± 3.8	15.4 ± 5.3	16.0 ± 6.5
Mean perfusion (%)	13.3 ± 4.2	13.5 ± 4.2	12.2 ± 3.0	14.3 ± 5.9	15.2 ± 5.6	14.4 ± 7.0
VDP (%)	13.1 ± 5.6	11.1 ± 6.0	8.4 ± 5.3	10.1 ± 8.5	11.6 ± 5.3	12.1 ± 7.3
QDP (%)	2.2 ± 3.6	1.6 ± 1.7	2.9 ± 5.9	2.2 ± 4.4	2.5 ± 4.5	1.5 ± 1.5
V/Qmatch_defected_ (%)	0.04 ± 0.09	0.2 ± 0.2	0.1 ± 0.2	0.4 ± 1.2	0.2 ± 0.6	0.2 ± 0.8
V/Qmatch_non−defected_ (%)	84.7 ± 6.1	87.5 ± 7.1	88.8 ± 9.0	88.1 ± 10.6	86.1 ± 7.6	86.6 ± 7.7

Unless otherwise specified, data are means ± SDs

*QDP* Perfusion defect percentage, *VDP* Ventilation defect percentage, *V/Qmatch *Ventilation-perfusion match, *PCS* Post-COVID-19 syndrome, *PREFUL MRI* Phase-resolved functional lung low-field magnetic resonance imaging

* Adjusted P-values were calculated using the Wilcoxon matched-pairs signed rank test to compare baseline and follow-up. Asterisks represent significant differences between baseline and follow-up. *P ≤ 0.05

### Post-hoc comparison to historic data

Compared to historic data [29], perfusion increased and QDP decreased across all cohorts, meaning controls (historic: healthy; baseline and follow-up: vaccinated), recovered and PCS (Supplementary Figs. 3 and 5). Ventilation showed no differences across cohorts, while VDP decreased for recovered and PCS (Supplementary Figs. 2 and 4). V/Qmatch_non−defected_ increased for recovered (historic: 63.5 ± 18.7%; baseline: 88.8 ± 9.0%, *p* < 0.0001; follow-up: 88.1 ± 10.6%; *p* = 0.0002) and PCS (historic: 57.3 ± 19.5%; baseline: 86.1 ± 7.6%, *p* < 0.0001; follow-up: 86.6 ± 7.7%, *p* = 0.0110) (Supplementary Fig. 7), while V/Qmatch_defected_ decreased accordingly in both recovered and PCS (Supplementary Fig. 6, Supplementary Table 3).

A subset of *n* = 3 PCS and *n* = 5 recovered who were followed through both studies showed similar mean ventilation. The subset showed increased mean perfusion and V/Qmatch_non−defected_ between the first and third timepoints. VDP decreased in this subset, as did the QDP and V/Qmatch_defected_ (Fig. 3a and b, Supplementary Table 4).

## Discussion

The aim of this study was to assess lung function parameters in children and adolescents in the context of SARS-CoV-2 infection using PREFUL MRI. At baseline and at 6-month follow-up, we found a low degree of functional abnormalities with general trends of improvement between timepoints. In comparison with historic data [29], we found relevant improvement of almost all measured parameters, indicating a widespread clinical recovery from early virus variant infection.

Although functional lung impairments and altered pulmonary diffusion capacity [29], ventilation/perfusion mismatch and hyperventilation syndrome have been documented [37], our follow-up examinations provide evidence of good recovery without differences in clinical subgroups. Persistent small airway dysfunction and lung hyperinflation were described in adults [38], which was also shown in various imaging procedures [39, 40]. However, changes in computed tomography could not be confirmed in children [41], which is also supported by this study over a long-term follow-up.

Apart from PREFUL MRI, ^129^Xe MRI has been widely used for evaluating lung injuries caused by asthma [42], chronic obstructive pulmonary disease [43], cystic fibrosis [44], idiopathic pulmonary fibrosis [45], and COVID-19 [25, 26, 28, 46, 47]. Using ^129^Xe MRI, similar longitudinal improvements in persisting lung impairment were previously observed during follow-up of patients affected by PCS, with some defects remaining 1-year post-infection [47]. Improvement in VDP from 3 months to 15 months post-infection has been shown using ^129^Xe MRI [28], matching the trend in our findings, although we found smaller differences, potentially because the time between infection and baseline was longer. In adults hospitalized due to COVID, lower ^129^Xe MRI parameters were found than in controls about eight months after infection [25], as well as lower ^129^Xe MRI VDP [26]. Given a low hospitalization rate in children, we could not observe similar findings. Similarly to PREFUL MRI, ^129^Xe MRI has proven advantageous for longitudinal studies involving children due to the absence of radiation [48]. However, ^129^Xe MRI requires the active inhalation of a xenon gas, whereas PREFUL MRI does not require any additional substances. Like our imaging results, spirometry and body plethysmography were found to be normal in children with PCS [12, 15].

Our study had several limitations. First, we had fewer vaccinated and recovered controls and the predetermined sample size couldn’t be reached due to the low number of participating families. Secondly, there is a selection bias because families with children suffering from PCS were more likely to participate in our study, whereas those with acute or post-acute PCS and potentially even higher disease burden might have been deterred by the number of examinations required by the superordinate study, and consequently were more likely to be lost to follow-up. At least one case of a participant unwilling to complete follow-up due to likely post-exertional malaise (PEM) is known to the authors [49]. Severe forms of PCS with PEM are seen as overlap with myalgic encephalomyelitis / chronic fatigue syndrome (ME/CFS) [50], meaning our results could underestimate the severity relative to the population mean. Since older children and adolescents are more likely to be diagnosed with PCS and were more likely to participate in our study [51], our results could overestimate the severity compared to the population mean. Thirdly, since we focused primarily on physical parameters, the trial design was limited in its ability to assess psychological and psychosomatic factors. Fourth, our study is limited by its single-center design, due to the experimental nature and limited availability of the novel imaging modality used. Fifth, regarding the three timepoint comparison, the cohort was small and therefore statistically limited. The results, while significant, need further reevaluation in larger comparisons.

In summary, our study demonstrates the recovery of pulmonary function after SARS-CoV-2 infection as visualized by PREFUL MRI in both children and adolescents.

When compared to historic data of early SARS-CoV-2 variants infections, we currently observe an absence of relevant functional lung impairments in our study cohort.

PREFUL MRI appears to be suitable for longitudinal assessment of lung function of children affected by COVID-19.

## Supplementary Information

Supplementary Material 1.

## Data Availability

The datasets used and/or analyzed during the current study are available from the corresponding author on reasonable request.

## Abbreviations

CRP: c-reactive protein (CRP)
IL-6: Interleukin-6 (IL-6)
LF- MRI: Low-field magnetic resonance imaging
ME/CFS: Myalgic encephalomyelitis / chronic fatigue syndrome
PCC: Post-COVID-19 condition
PCS: Post-COVID-19 syndrome
PEM: Post-exertional malaise
PREFUL MRI: Phase-resolved functional magnetic resonance lung imaging
Q: Perfusion
QDP: Perfusion defects
V: Ventilation
VDP: Ventilation Defects
V/Qmatch_non-defected_, V/Qmatch_defected_: Match/non-match of ventilation and perfusion
