# Supplementary material for: Longitudinal functional lung imaging in children with Post-COVID-19 syndrome
Source: Mol Cell Pediatr. 2026 Jan 31;13:4. doi: 10.1186/s40348-025-00216-x (PMC12858673; doi:10.1186/s40348-025-00216-x)
Supplement: Supplementary file 1 — Supplementary Material 1. [file 40348_2025_216_MOESM1_ESM.docx]

**Supplementary Appendix**

Supplement to: Kraus C et.al. Longitudinal functional lung imaging in children with Post-COVID-19 syndrome

This appendix has been provided by the authors to give readers additional information about the work.

Content

[Supplementary Material and Methods 3](#_Toc178917853)

[Supplementary Figures 5](#_Toc178917854)

[Supplementary Tables 13](#_Toc178917855)

[References 18](#_Toc178917856)

# Supplementary Material and Methods

**Clinical data and blood samples**

As described in the preliminary study [1], the same protocol was followed when analyzing the blood samples. All blood samples were centrifuged and stored as serum at −15°C. Complete blood counts were done using Sysmex XE-2100 (Sysmex, Kobe, Japan) analyzers, serum interleukin-6 (IL-6) was measured by electrochemiluminescence immunoassay (Elecsys IL-6, Cobas e601) and C-reactive protein (CRP) was measured using a standard immunoturbidometric assay on the Cobas c501 system (Roche Diagnostics).

For qualitative testing of antibodies against SARS-CoV-2, an electrochemiluminescence immunoassay (ECLIA), the Elecsys Anti-SARS-CoV-2 assay, was used. As it utilizes a recombinant protein representing the nucleocapsid (N) antigen in a double-antigen sandwich assay format, all antibody isotypes (IgM, IgA, IgG) are detected. According to the manufacturer’s cut-off index test results above 1.0 were reactive and interpreted as confirmed contact with wild-type SARS-CoV-2 virus. The assay was evaluated to have a sensitivity of 99.5% 14 days after the first positive RT-PCR results (95% CI: 97.0%–100%) and a specificity of 99.8% (95% CI: 99.69%-99.88%) [2].

For quantitative determination of antibodies to the SARS-CoV-2 spike (S) protein a Cobas 601 module with the Elecsys Anti-SARS-CoV-2-S immunoassay (ACOV2S) was used. By using a double-antigen sandwich immunoassay a recombinant protein representing the receptor binding domain of the spike protein enables the identification of highly affine SARS-CoV-2 IgM and IgG antibodies. Test results above 0.8 U/mL were considered reactive, following the manufacturer’s cut-off-index and interpreted as a confirmed immunological response to either SARS-CoV-2 virus or mRNA vaccines. Values between 0.40–250 U/mL represent the linear range. Samples above 250 U/mL were automatically diluted into the linear range of the assay (realized dilutions: 1:10 or 1:100) with Diluent Universal (Roche Diagnostics). Diluted results were automatically multiplied by the dilution factor, which enabled an upper limit of quantification of 25,000 U/mL. The assay demonstrated a sensitivity of 98.9% (95% CI: 98.1%–99.3%) 14 days after the first positive RT-PCR result and a specificity of 99.96% (95% CI: 99.91%–100%) [3]. Since the assigned U/mL are equivalent to Binding Antibody Units (BAU)/mL as defined by the first World Health Organization (WHO) International Standard for anti-SARS-CoV-2 immunoglobulin (NIBSC code 20/136), our results can be directly compared with other studies or results in BAU/mL without the need for conversion of units.

# Supplementary Figures


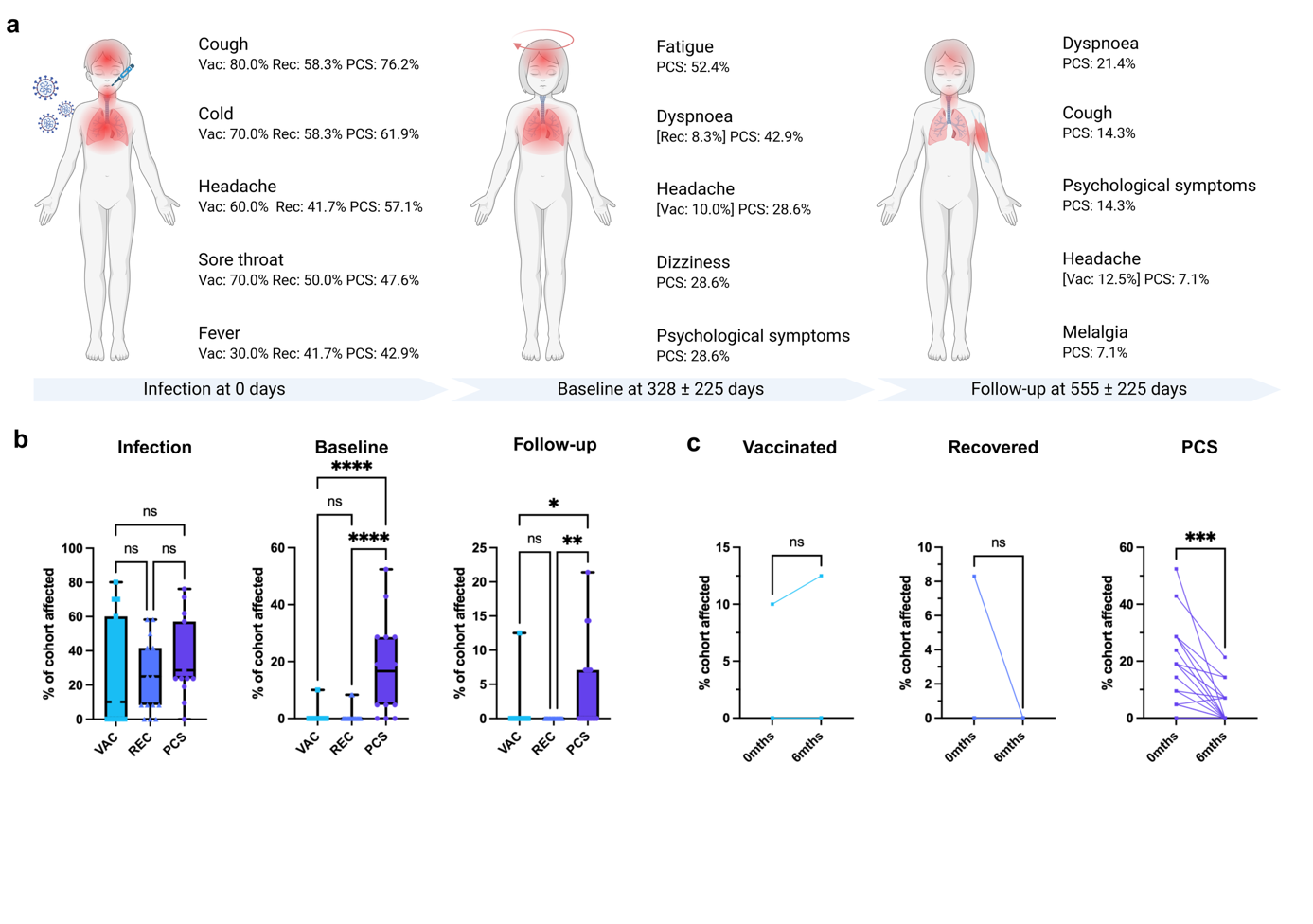


**Supplementary Figure 1 – Clinical symptoms of Post-COVID-19 syndrome patients during the study**

**(a)** Illustration of the five most common symptoms reported at initial infection, at baseline (0 months) and at follow-up (about six months) later. Given days with standard deviations are days since initial infection. Note: two participants in recovered reported persistent dyspnea or headache, despite stating not to suffer from prolonged symptoms. 1 vaccinated reported persisting headache after infection and vaccination. Data are percentages of the cohort affected by each symptom. For the vaccinated controls, only those with previous infection were included. Vac = Vaccinated; Rec = Recovered; PCS = Post-COVID-19 syndrome

**(b)** Percentage of cohorts affected by any specific symptom at time of initial infection, at baseline and at 6-month follow-up. Comparisons across cohorts were made using Kruskal-Wallis test. Primary symptoms include headache, cold, sore throat, cough, dyspnea, pneumonia, fever, loss of scent and taste, fatigue and melalgia. Secondary symptoms include dizziness; gastrointestinal, neurological, and psychological symptoms; susceptibility to infection and inflammation; and other symptoms. Asterisks represent significant differences. *P ≤ 0.05, **P ≤ 0.01, ***P ≤ 0.001, ****P ≤ 0.0001

**(c)** Percentage of cohorts affected by any specific symptom compared longitudinally between baseline and 6-month follow-up for each cohort. Primary symptoms include headache, cold, sore throat, cough, dyspnea, pneumonia, fever, loss of scent and taste, fatigue and melalgia. Secondary symptoms include dizziness; gastrointestinal, neurological, and psychological symptoms; susceptibility to infection and inflammation; and other symptoms. Statistical analysis was performed using the Wilcoxon matched-pairs signed rank test. Asterisks represent significant differences. *P ≤ 0.05, **P ≤ 0.01, ***P ≤ 0.001, ****P ≤ 0.0001


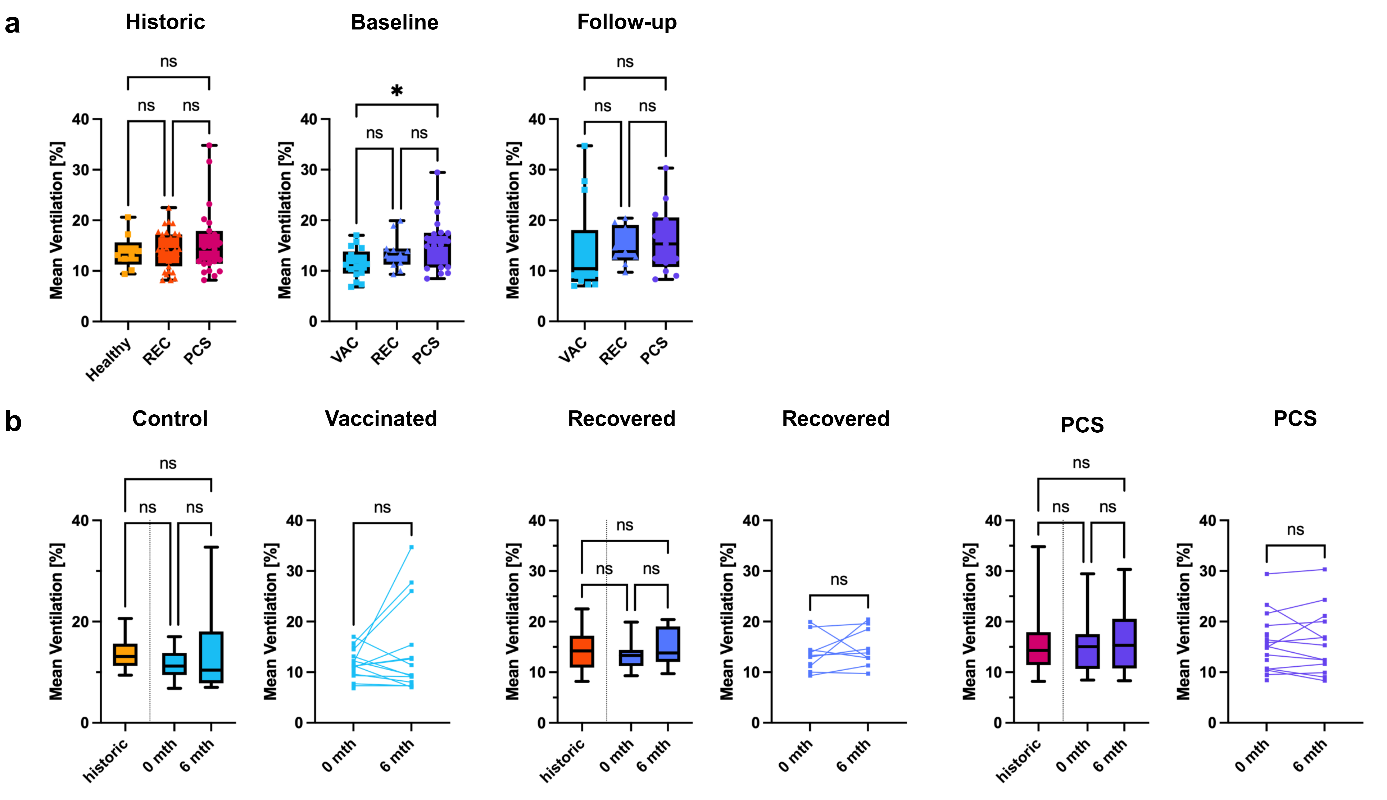


**Supplementary Figure 2 – PREFUL MRI: Mean ventilation compared by cohort and timepoints**

**(a**) Mean ventilation derived from PREFUL MRI, including historic data, compared by timepoint using Kruskal-Wallis test [1].

**(b)** Mean ventilation compared longitudinally by cohort including historic data. Control consisted of healthy participants in the historic dataset and vaccinated individuals at baseline and follow-up six months later. Analyses including historic were performed using Kruskal-Wallis test, while comparison between baseline and follow-up were done using Wilcoxon matched-pairs signed rank test.

Vac = Vaccinated, Rec = Recovered, PCS = Post-COVID-19 syndrome, PREFUL MRI = Phase-resolved functional lung low-field magnetic resonance imaging

Asterisks represent significant differences. *P ≤ 0.05, **P ≤ 0.01, ***P ≤ 0.001, ****P ≤ 0.0001


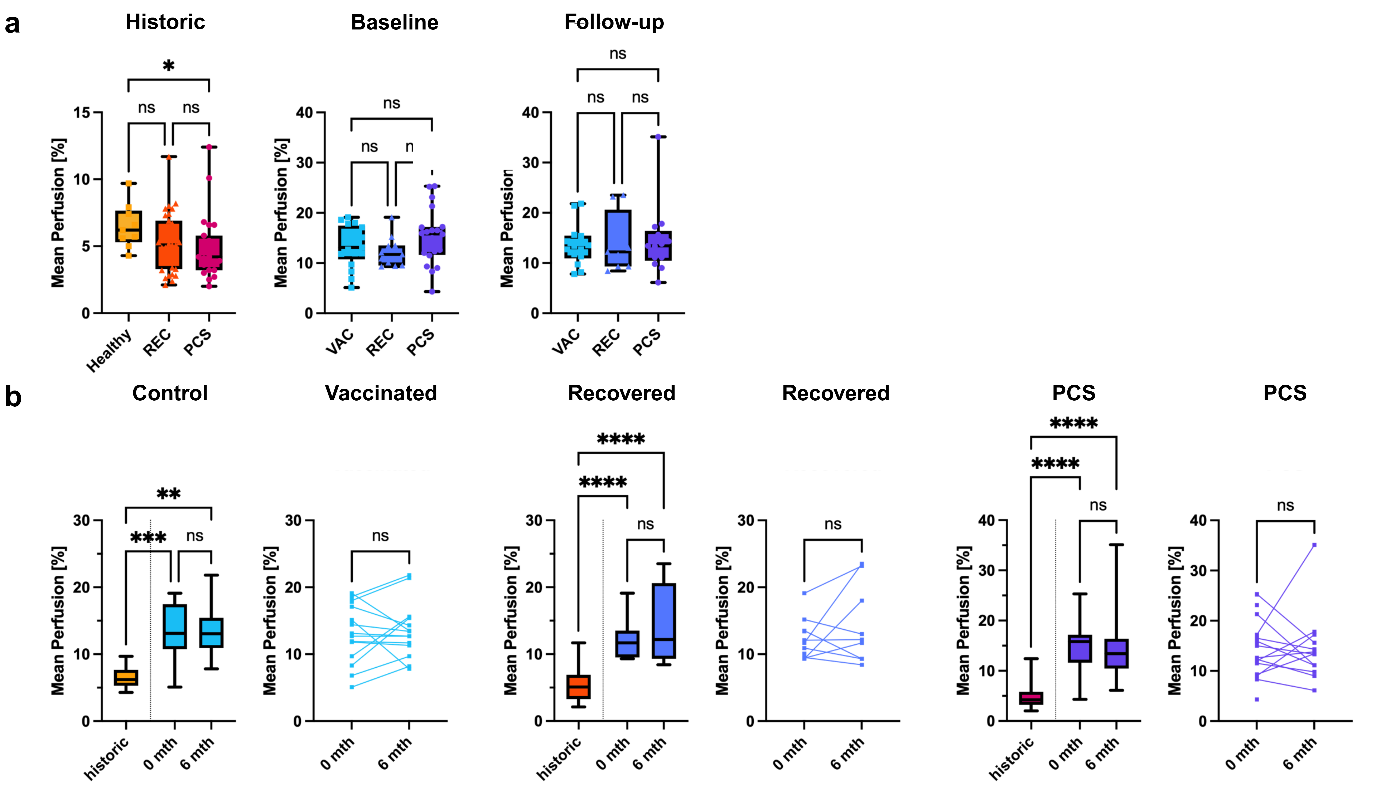


**Supplementary Figure 3 – PREFUL MRI: Mean perfusion compared by cohort and timepoints**

**(a**) Mean perfusion derived from PREFUL MRI, including historic data, compared by timepoint using Kruskal-Wallis test [1].

**(b)** Mean perfusion compared longitudinally by cohort including historic data. Control consisted of healthy participants in the historic dataset and vaccinated individuals at baseline and follow-up six months later. Analyses including historic were performed using Kruskal-Wallis test, while comparison between baseline and follow-up were done using Wilcoxon matched-pairs signed rank test.

Vac = Vaccinated, Rec = Recovered, PCS = Post-COVID-19 syndrome, PREFUL MRI = Phase-resolved functional lung low-field magnetic resonance imaging

Asterisks represent significant differences. *P ≤ 0.05, **P ≤ 0.01, ***P ≤ 0.001, ****P ≤ 0.0001


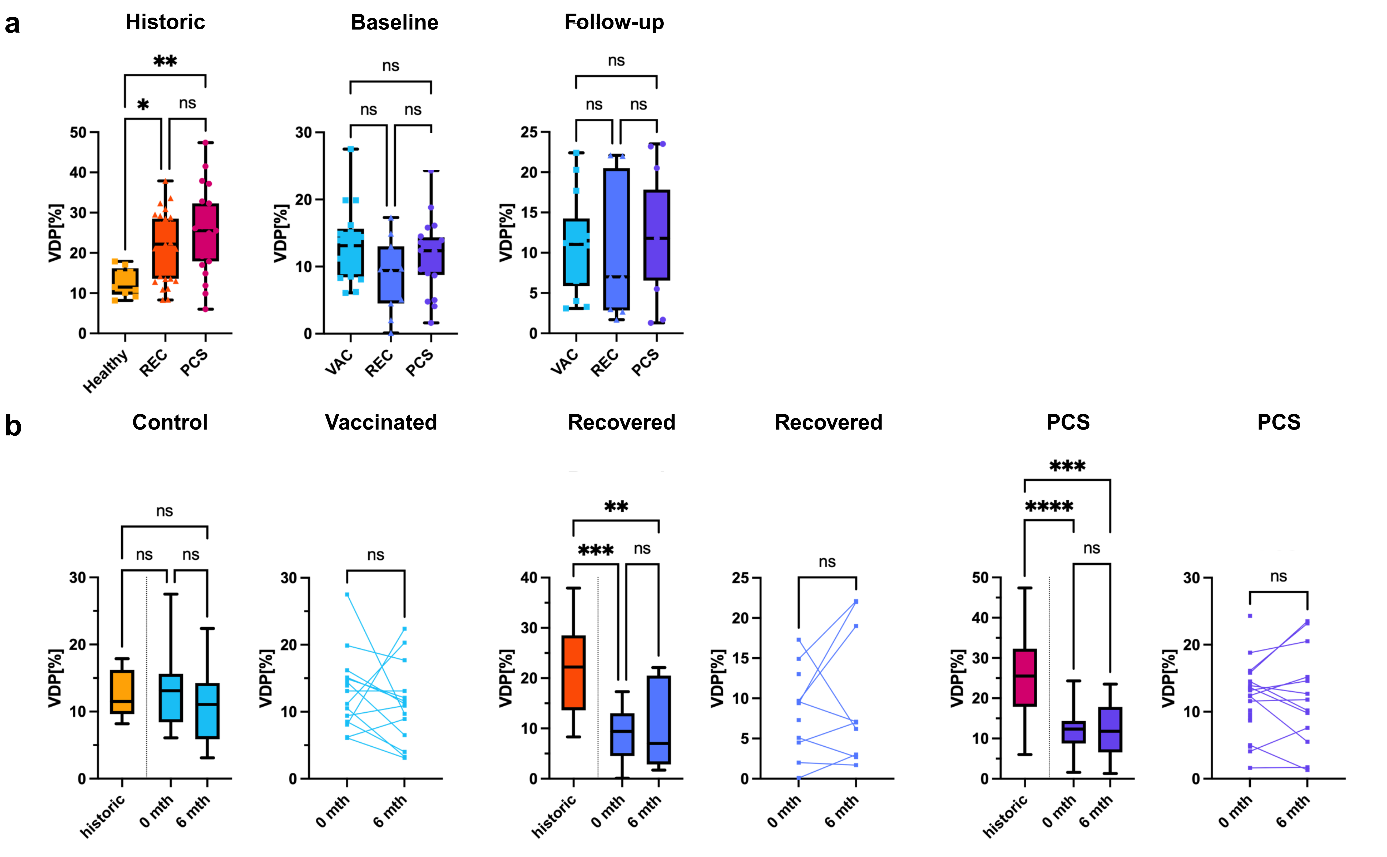


**Supplementary Figure 4 – PREFUL MRI: VDP compared by cohort and timepoints**

**(a**) Ventilation defect percentage (VDP) derived from PREFUL MRI, including historic data, compared by timepoint using Kruskal-Wallis test [1].

**(b)** VDP compared longitudinally by cohort including historic data. Control consisted of healthy participants in the historic dataset and vaccinated individuals at baseline and follow-up six months later. Analyses including historic were performed using Kruskal-Wallis test, while comparison between baseline and follow-up were done using Wilcoxon matched-pairs signed rank test.

Vac = Vaccinated, Rec = Recovered, PCS = Post-COVID-19 syndrome, PREFUL MRI = Phase-resolved functional lung low-field magnetic resonance imaging, VDP = Ventilation defect percentage

Asterisks represent significant differences. *P ≤ 0.05, **P ≤ 0.01, ***P ≤ 0.001, ****P ≤ 0.0001


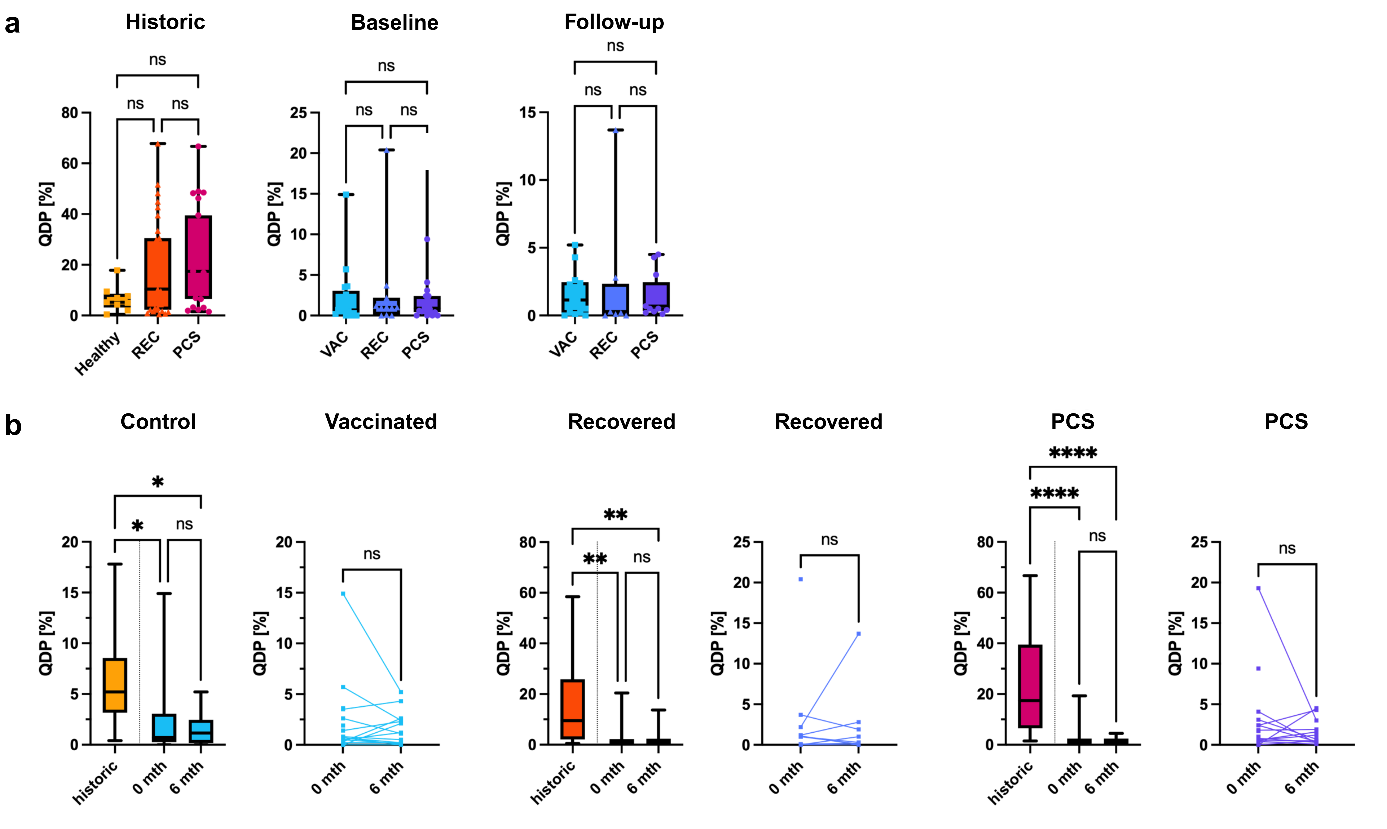


**Supplementary Figure 5 – PREFUL MRI: QDP compared by cohort and timepoints**

**(a**) Perfusion defect percentage (QDP) derived from PREFUL MRI, including historic data, compared by timepoint using Kruskal-Wallis test [1].

**(b)** QDP compared longitudinally by cohort including historic data. Control consisted of healthy participants in the historic dataset and vaccinated individuals at baseline and follow-up six months later. Analyses including historic were performed using Kruskal-Wallis test, while comparison between baseline and follow-up were done using Wilcoxon matched-pairs signed rank test.

Vac = Vaccinated, Rec = Recovered, PCS = Post-COVID-19 syndrome, PREFUL MRI = Phase-resolved functional lung low-field magnetic resonance imaging, QDP = Perfusion defect percentage

Asterisks represent significant differences. *P ≤ 0.05, **P ≤ 0.01, ***P ≤ 0.001, ****P ≤ 0.0001


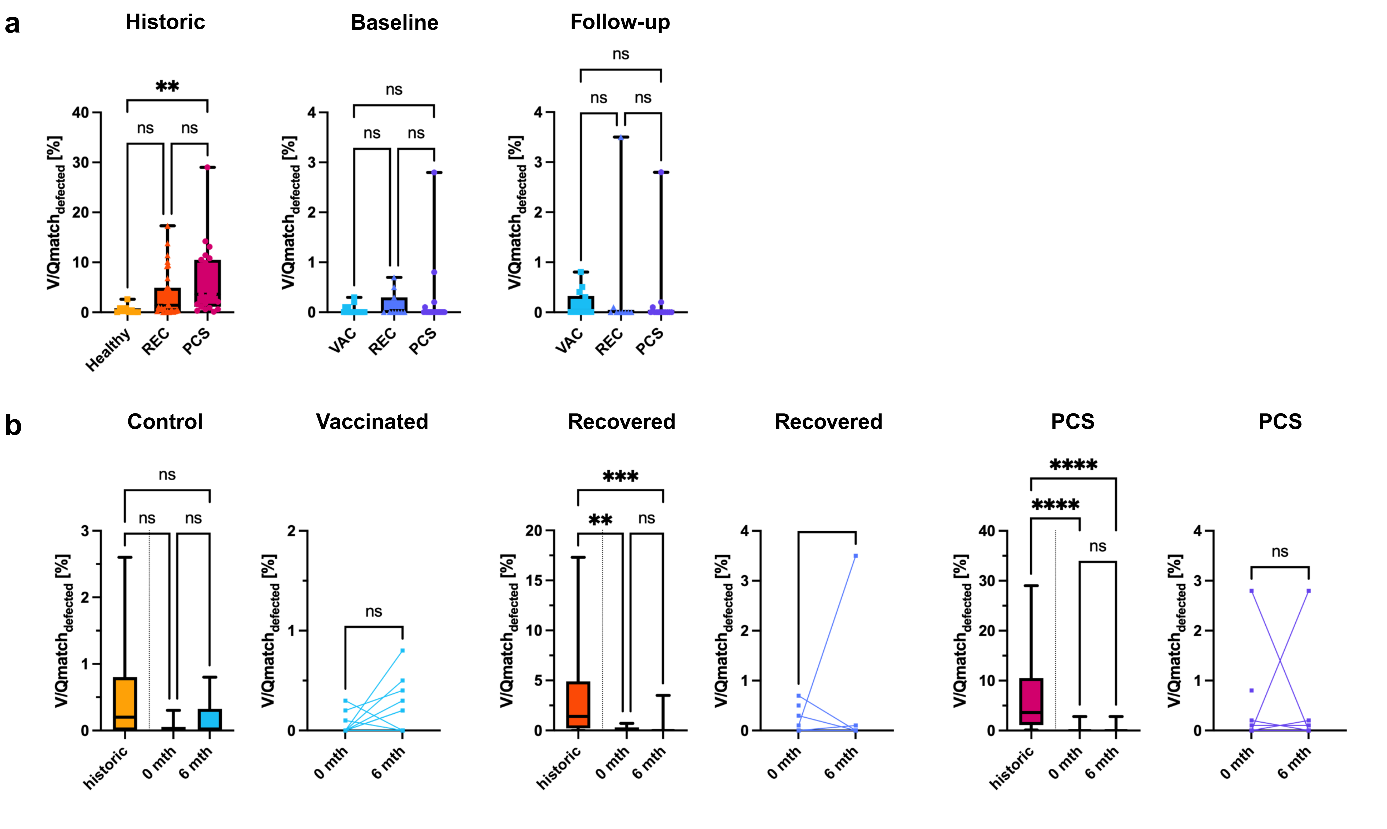


**Supplementary Figure 6 – PREFUL MRI: V/Qmatch_defected_ compared by cohort and timepoints**

**(a**) V/Qmatch_defected_ derived from PREFUL MRI, including historic data, compared by timepoint using Kruskal-Wallis test [1].

**(b)** V/Qmatch_defected_ compared longitudinally by cohort including historic data. Control consisted of healthy participants in the historic dataset and vaccinated individuals at baseline and follow-up six months later. Analyses including historic were performed using Kruskal-Wallis test, while comparison between baseline and follow-up were done using Wilcoxon matched-pairs signed rank test.

Vac = Vaccinated, Rec = Recovered, PCS = Post-COVID-19 syndrome, PREFUL MRI = Phase-resolved functional lung low-field magnetic resonance imaging, V/Qmatch_defected_ = Ventilation-perfusion match of defected areas

Asterisks represent significant differences. *P ≤ 0.05, **P ≤ 0.01, ***P ≤ 0.001, ****P ≤ 0.0001


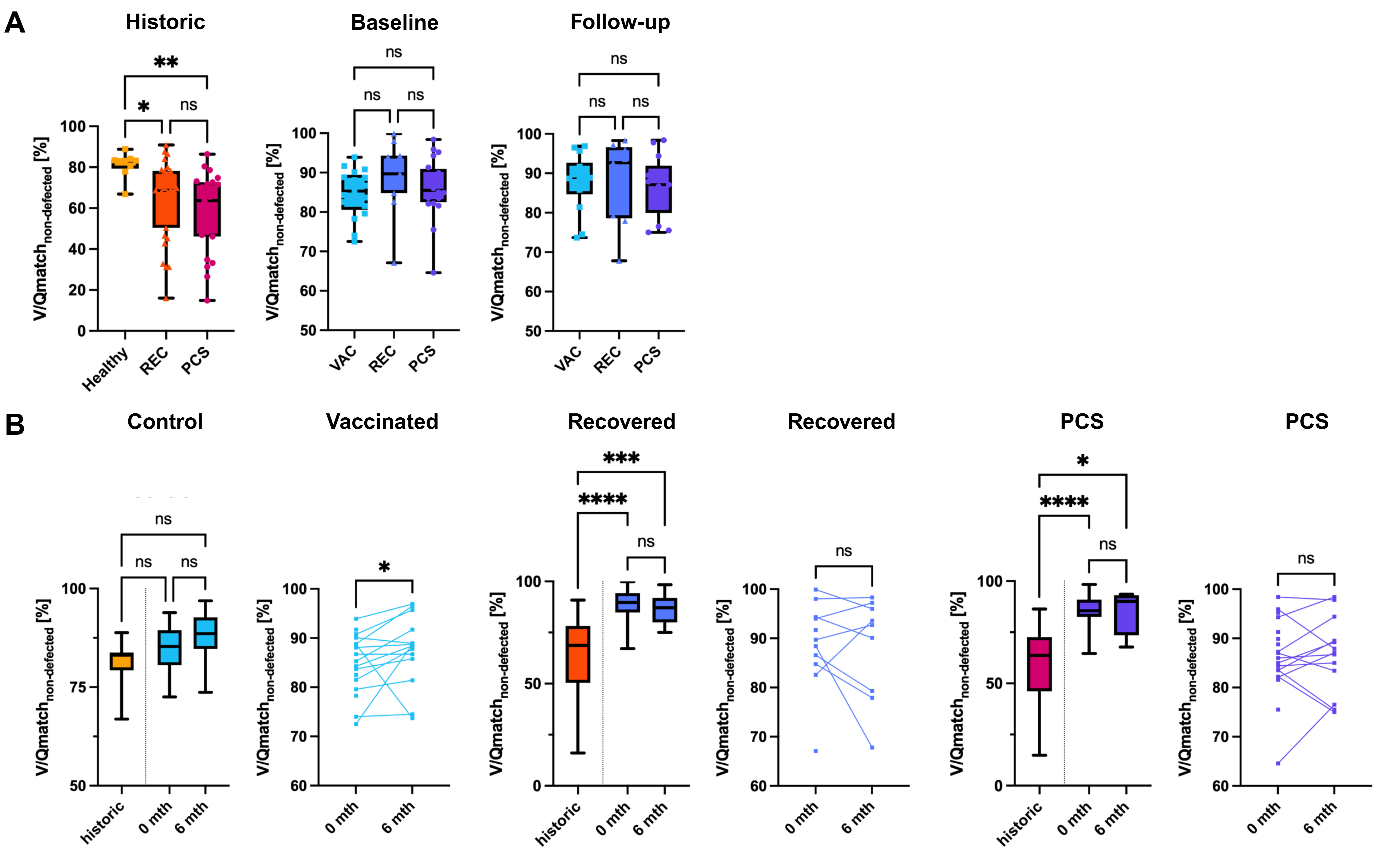


**Supplementary Figure 7 – PREFUL MRI: V/Qmatch_non-defected_ compared by cohort and timepoints**

**(a**) V/Qmatch_non-defected_ derived from PREFUL MRI, including historic data, compared by timepoint using Kruskal-Wallis test [1].

**(b)** V/Qmatch_non-defected_ compared longitudinally by cohort including historic data. Control consisted of healthy participants in the historic dataset and vaccinated individuals at baseline and follow-up six months later. Analyses including historic were performed using Kruskal-Wallis test, while comparison between baseline and follow-up were done using Wilcoxon matched-pairs signed rank test.

Vac = Vaccinated, Rec = Recovered, PCS = Post-COVID-19 syndrome, PREFUL MRI = Phase-resolved functional lung low-field magnetic resonance imaging, V/Qmatch_non-defected_ = Ventilation-perfusion match of non-defected areas

Asterisks represent significant differences. *P ≤ 0.05, **P ≤ 0.01, ***P ≤ 0.001, ****P ≤ 0.0001

# Supplementary Tables

**Supplementary Table 1 - Clinical symptoms**

|  | **Vaccinated** | | | **Recovered** | | | **PCS** | | |
| --- | --- | --- | --- | --- | --- | --- | --- | --- | --- |
| **Examination** | **Infection (n=10)** | **Baseline**  **(n=10)** | **Follow-up**  **(n=8)** | **Infection (n=12)** | **Baseline**  **(n=12)** | **Follow-up**  **(n=11)** | **Infection (n=21)** | **Baseline**  **(n=21)** | **Follow-up**  **(n=14)** |
| **Primary symptoms** |  |  |  |  |  |  |  |  |  |
| Headache | 6 (60.0) | 1 (10.0) | 1 (12.5) | 5 (41.7) | 0 (0) | 0 (0) | 12 (57.1) | 6 (28.6) | 1 (7.1) |
| Cold | 7 (70.0) | 0 (0) | 0 (0) | 7 (58.3) | 0 (0) | 0 (0) | 13 (61.9) | 0 (0) | 0 (0) |
| Sore throat | 7 (70.0) | 0 (0) | 0 (0) | 6 (50.0) | 0 (0) | 0 (0) | 10 (47.6) | 4 (19.0) | 0 (0) |
| Cough | 8 (80.0) | 0 (0) | 0 (0) | 7 (58.3) | 0 (0) | 0 (0) | 16 (76.2) | 4 (19.0) | 2 (14.3) |
| Dyspnoea | 0 (0) | 0 (0) | 0 (0) | 1 (8.3) | 1 (8.3) | 0 (0) | 7 (33.3) | 9 (42.9) | 3 (21.4) |
| Pneumonia | 0 (0) | 0 (0) | 0 (0) | 0 (0) | 0 (0) | 0 (0) | 0 (0) | 0 (0) | 0 (0) |
| Fever | 3 (30.0) | 0 (0) | 0 (0) | 5 (41.7) | 0 (0) | 0 (0) | 9 (42.9) | 1 (4.8) | 0 (0) |
| Loss of scent | 1 (10.0) | 0 (0) | 0 (0) | 4 (33.3) | 0 (0) | 0 (0) | 5 (23.8) | 2 (9.5) | 0 (0) |
| Loss of taste | 0 (0) | 0 (0) | 0 (0) | 3 (25.0) | 0 (0) | 0 (0) | 5 (23.8) | 0 (0) | 0 (0) |
| Fatigue | 3 (30.0) | 0 (0) | 0 (0) | 3 (25.0) | 0 (0) | 0 (0) | 15 (71.4) | 11 (52.4) | 0 (0) |
| Melalgia | 1 (10.0) | 0 (0) | 0 (0) | 1 (8.3) | 0 (0) | 0 (0) | 6 (28.6) | 4 (19.0) | 1 (7.1) |
| **Secondary symptoms** |  |  |  |  |  |  |  |  |  |
| Dizziness | 0 (0) | 0 (0) | 0 (0) | 1 (8.3) | 0 (0) | 0 (0) | 4 (19.0) | 6 (28.6) | 0 (0) |
| Gastrointestinal | 3 (30.0) | 0 (0) | 0 (0) | 1 (8.3) | 0 (0) | 0 (0) | 5 (23.8) | 1 (4.8) | 0 (0) |
| Neurological | 0 (0) | 0 (0) | 0 (0) | 0 (0) | 1 (8.3) | 0 (0) | 5 (23.8) | 5 (23.8) | 0 (0) |
| Psychological | - | 0 (0) | 0 (0) | - | 0 (0) | 0 (0) | - | 6 (28.6) | 2 (14.3) |
| Susceptibility to infection | - | 0 (0) | 0 (0) | - | 0 (0) | 0 (0) | - | 2 (9.5) | 1 (7.1) |
| Susceptibility to inflammation | - | 0 (0) | 0 (0) | - | 0 (0) | 0 (0) | - | 1 (4.8) | 1 (7.1) |
| Others | 0 (0) | 0 (0) | 0 (0) |  | 0 (0) | 0 (0) | 2 (9.5) | 3 (14.3) | 0 (0) |
| **Symptom load^a^** | 26.0 ± 30.0 | 0.6 ± 2.4 | 0.7 ± 2.9 | 24.4 ± 21.5 | 0.9 ± 2.7 | 0.0 ± 0.0 | 36.2 ± 22.5 | 17.2 ± 15.0 | 4.4 ± 6.5 |

**Supplementary Table 1 - Clinical symptoms**

Unless otherwise specified, data are numbers of participants, with percentages in parentheses.

Diarrhea, nausea and vomiting and abdominal pain were clustered as gastrointestinal symptoms; sight and sleeping disorder, tinnitus, paresthesia, phantosmia, absence seizure and aphasia as neurological symptoms; depression, forgetfulness and weak concentration as psychological symptoms and undefined chest pain, sensitivity to low temperatures, cramping fingers and recurring hematoma as other symptoms. Symptom load represents the average proportion of the cohorts affected by the specific predefined symptoms or clusters.

^a^ Data are means ± SDs.

**Supplementary Table 2 - Laboratory Assessments**

|  | **Vaccinated** |  | **Recovered** |  | **PCS** |  |
| --- | --- | --- | --- | --- | --- | --- |
|  | **Baseline** | **Follow-up** | **Baseline** | **Follow-up** | **Baseline** | **Follow-up** |
| Hemoglobin (g/dL) | 13.3 ± 0.9 | 13.4 ± 1.0 | 13.7 ± 0.7 | 13.7 ± 0.9 | 13.2 ± 0.9 | 13.6 ± 1.0 |
| Thrombocytes (x 10^3^/µl) | 272 ± 60 | 290 ± 81 | 318 ± 78 | 264 ± 80 | 281 ± 68 | 279 ± 60 |
| Leukocytes (x 10^3^/µl) | 5.9 ± 1.5 | 6.3 ± 1.2 | 7.5 ± 2.1 | 6.9 ± 2.1 | 7.5 ± 2.3 | 7.1 ± 1.8 |
| C-reactive protein (mg/L) | 0.2 ± 0.7 | 0.4 ± 1.1 | 0.0 ± 0.0 | 0.2 ± 0.3 | 0.5 ± 1.1 | 0.7 ± 1.7 |
| Interleukin-6 (pg/mL) | 0.3 ± 0.9 | 1.1 ± 1.4 | 1.0 ± 1.2 | 2.2 ± 1.9 | 2.0 ± 5.7 | 0.8 ± 1.2 |
| **SARS-CoV-2 serostatus** |  |  |  |  |  |  |
| Nucleocapsid antibody (U/mL) | 33.3 ± 40.6 |  | 71.8 ± 82.3 |  | 65.9 ± 72.0 |  |
| Nucleocapsid antibody seropositive particpants^a^ | 11 (84.6) |  | 9 (100) |  | 14 (100) |  |
| Spike protein antibody (U/mL) | 12682 ± 8342 |  | 9122 ± 11584 |  | 6626 ± 6824 |  |
| Spike protein antibody seropositive participants^a^ | 13 (100) |  | 9 (100) |  | 14 (100) |  |

**Supplementary Table 2 - Laboratory Assessments**

Unless otherwise specified, data are means ± SDs. Unities are given in parenthesis.

^a^ Data are numbers of participants, with percentages in parentheses.

**Supplementary Table 3 - PREFUL MRI parameters including historic**

|  | **Control/Vaccinated** | | | **Recovered** | | | **PCS** | | | |
| --- | --- | --- | --- | --- | --- | --- | --- | --- | --- | --- |
| **Examination** | **Historic**  **(n=9)** | **Baseline (n=17)** | **Follow-up (n=14)** | **Historic**  **(n=31)** | **Baseline (n=11)** | **Follow-up (n=9)** | **Historic**  **(n=23)** | **Baseline (n=20)** | **Follow-up (n=13)** |  |
| Mean ventilation (%) | 13.7 ± 3.4 | 11.5 ± 2.9  p=ns | 14.1 ± 8.8  p=ns | 14.2 ± 3.8 | 13.6 ± 3.3  p=ns | 14.8 ± 3.8  p=ns | 15.6 ± 6.8 | 15.4 ± 5.3  p=ns | 16.0 ± 6.5  p=ns |  |
| Mean perfusion (%) | 6.5 ± 1.6 | 13.3 ± 4.2  p=0.0006 | 13.5 ± 4.2  p=0.0011 | 5.3 ± 2.2 | 12.2 ± 3.0  p<0.0001 | 14.3 ± 5.9  p<0.0001 | 4.9 ± 2.4 | 15.2 ± 5.6  p<0.0001 | 14.4 ± 7.0  p<0.0001 |  |
| VDP (%) | 12.8 ± 3.6 | 13.1 ± 5.6  p=ns | 11.1 ± 6.0  p=ns | 21.8 ± 7.9 | 8.4 ± 5.3  p=0.0002 | 10.1 ± 8.5  p=0.0026 | 25.2 ± 10.2 | 11.6 ± 5.3  p<0.0001 | 12.1 ± 7.3  p=0.0009 |  |
| QDP (%) | 6.5 ± 5.0 | 2.2 ± 3.6  p=0.0151 | 1.6 ± 1.7  p=0.0156 | 18.3 ± 18.5 | 2.9 ± 5.9  p=0.0044 | 2.2 ± 4.4  p=0.0021 | 23.4 ± 19.0 | 2.5 ± 4.5  p<0.0001 | 1.5 ± 1.5  p<0.0001 |  |
| V/Qmatch_defected_ (%) | 0.5 ± 0.8 | 0.04 ± 0.09  p=ns | 0.2 ± 0.2  p=ns | 3.6 ± 4.6 | 0.1± 0.2  p=0.0011 | 0.4 ± 1.2  p=0.0010 | 5.9 ± 6.7 | 0.2 ± 0.6  p<0.0001 | 0.2 ± 0.8  p<0.0001 |  |
| V/Qmatch_non-defected_ (%) | 81.2 ± 6.1 | 84.7 ± 6.1  p=ns | 87.5 ± 7.1  p=ns | 63.5 ± 18.7 | 88.8 ± 9.0  p<0.0001 | 88.1 ± 10.6  p=0.0002 | 57.3 ± 19.5 | 86.1 ± 7.6  p<0.0001 | 86.6 ± 7.7  p=0.0110 |  |

**Supplementary Table 3 - PREFUL MRI parameters including historic**

Unless otherwise specified, data are means ± SDs. The control group consists of healthy participants for historic and vaccinated children and adolescents for baseline and follow-up.

Adjusted P-values were tested with Kruskal-Wallis test and corrected Dunn test for post hoc comparisons between timepoints and given in comparison with historic data. Using Kruskal-Wallis test no difference was found between baseline and follow-up.

QDP = Perfusion defect percentage, VDP = Ventilation defect percentage, V/Qmatch = Ventilation-perfusion match, PREFUL MRI = Phase-resolved functional lung low-field magnetic resonance imaging

**Supplementary Table 4 - PREFUL MRI – Comparison with historic data of a subset examined at 3 timepoints**

|  | **Recovered** | | | **PCS** | | |
| --- | --- | --- | --- | --- | --- | --- |
| **Examination** | **Historic (n=5)** | **Baseline (n=5)** | **Follow-up (n=5)** | **Historic (n=3)** | **Baseline (n=3)** | **Follow-up (n=2)** |
| Mean ventilation (%) | 13.2 ± 3.2 | 11.6 ± 2.2 | 13.6 ± 4.1 | 12.5 ± 2.9 | 14.4 ± 3.5 | 12.0 ± 0.6 |
| Mean perfusion (%) | 5.3 ± 1.5 | 11.4 ± 2.4 | 10.9 ± 2.0 | 3.7 ± 1.0 | 14.7 ± 2.3 | 14.5 ± 4.7 |
| VDP (%) | 22.1± 8.4 | 10.1 ± 4.6 | 10.1 ± 4.6 | 25.8 ± 5.1 | 11.4 ± 1.6 | 10.4 ± 6.9 |
| QDP (%) | 14.2 ± 17.4 | 1.6 ± 1.4 | 3.8 ± 5.7 | 26.6 ± 21.3 | 0.5 ± 0.1 | 0.9 ± 1.1 |
| V/Qmatch_defected_ (%) | 2.3 ± 4.4 | 0.1 ± 0.1 | 0.7 ± 1.6 | 5.1 ± 5.0 | 0.0 ± 0.1 | 0.1 ± 0.1 |
| V/Qmatch_non-defected_ (%) | 66.0 ± 19.2 | 88.3 ± 4.3 | 88.3 ± 11.0 | 52.7 ± 21.7 | 88.1 ± 1.6 | 88.9 ± 7.8 |
| Infection to examination (d) | 225 ± 170 | 663 ± 111 | 834 ± 134 | 326±95 | 708±124 | 867 ± 168 |

**Supplementary Table 4 - PREFUL MRI – Comparison with historic data of a subset examined at 3 timepoints**

Unless otherwise specified, data are means ± SDs.

Abbreviations: Perfusion defect percentage (QDP), Ventilation defect percentage (VDP), Ventilation-perfusion match (V/Qmatch), PREFUL MRI = Phase-resolved functional lung low-field magnetic resonance imaging

# References

1. Heiss R, Tan L, Schmidt S, Regensburger AP, Ewert F, Mammadova D, et al. Pulmonary Dysfunction after Pediatric COVID-19. Radiology. 2023;306(3):e221250. <https://doi.org/10.1148/radiol.221250>

2. Muench P, Jochum S, Wenderoth V, Ofenloch-Haehnle B, Hombach M, Strobl M, et al. Development and Validation of the Elecsys Anti-SARS-CoV-2 Immunoassay as a Highly Specific Tool for Determining Past Exposure to SARS-CoV-2. J Clin Microbiol. 2020;58(10). <https://doi.org/10.1128/jcm.01694-20>

3. Roche Diagnostics International AG. Fact sheet, ElecsysT Anti-SARS-CoV-2 S [Available from: <https://diagnostics.roche.com/content/dam/diagnostics/Blueprint/en/pdf/cps/Elecsys-Anti-SARS-CoV-2-S-factsheet-SEPT-2020-2.pdf>.

The graphs used in this supplement were made with BioRender. The unique URLs are given as follows:

Supplementary Figure 1: Created in BioRender. Kraus, C. (2025) https://BioRender.com/ezu4edf

Supplementary Figure 2: Created in BioRender. Kraus, C. (2025) https://BioRender.com/q977jko

Supplementary Figure 3: Created in BioRender. Kraus, C. (2025) https://BioRender.com/5raoslp

Supplementary Figure 4: Created in BioRender. Kraus, C. (2025) https://BioRender.com/fzmz0da

Supplementary Figure 5: Created in BioRender. Kraus, C. (2025) https://BioRender.com/fpw0bi8

Supplementary Figure 6: Created in BioRender. Kraus, C. (2025) https://BioRender.com/yuj1o7d

Supplementary Figure 7: Created in BioRender. Kraus, C. (2025) https://BioRender.com/jxs7zje
